# Working between systems: an umbrella review of care navigator roles and responsibilities

**DOI:** 10.3389/frhs.2025.1632307

**Published:** 2025-10-24

**Authors:** Shalini Wijekulasuriya, Leanne Wahlstrom, Suzanne Lewis, Zoi Triandafilidis, Christina Rojas, Nicholas Goodwin, Antonina Semkina, Annette Boaz, Caroline Norrie, Yvonne Zurynski

**Affiliations:** ^1^ Australian Institute of Health Innovation, Macquarie University, Sydney, NSW, Australia; ^2^ Faculty of Medicine, Health and Human Sciences, Macquarie University, Sydney, NSW, Australia; ^3^ Central Coast Research Institute, University of Newcastle, Gosford, NSW, Australia; ^4^ Central Coast Local Health District, Gosford, NSW, Australia; ^5^ NIHR Health and Social Care Workforce Research Unit, The Policy Institute, King’s College London, London, United Kingdom

**Keywords:** care navigator, care coordinator, health and social care, link worker, patient navigator, social prescribing, systematic review, workforce

## Abstract

**Background:**

A growing workforce is being employed internationally to assist patients in navigating between health and social care providers. These roles operate under various care models including patient navigation, social prescribing, and care coordination; tasks and responsibilities of care navigators are highly variable and often lack clarity. Understanding the similarities and differences amongst care navigator roles could improve the embedding of roles into health and social care systems and legitimise professional identity. This umbrella review synthesises evidence on care navigator roles, role titles, tasks, and functions, across diverse models that integrate care at the health and social care interface.

**Methods:**

MEDLINE, Embase, CINAHL, Scopus, and PsycINFO were searched from 1 Jan 2019–31 May 2024. Reviews that used systematic, scoping, or other rigorous methodology were included if they discussed the role or function of workers who coordinated services involving health and social care. Data were synthesised using an inductive thematic approach.

**Results:**

Twenty-six review articles were included, which synthesised 824 unique primary sources. Seventy-eight unique role titles were used to describe care navigators, which aggregated under seven role categories: *Patient Navigator*, *Link Worker*, *Care Coordinator*, *Case Manager*, *Social Prescriber*, *Intermediary*, *Health Mediator*. The most common were *Patient Navigator* and *Link Worker*. Tasks related to navigation, building service users' capacity for self-management, and providing person-centred care overlapped across all role categories, indicating the core functions of the navigation workforce. *Patient Navigators*' scope of practice included the provision of education, appointment coordination, and assistance with logistic issues, while the roles of *Link Workers* typically only provided referral-based navigation and developed the capacity of service users for self-management.

**Conclusions:**

The range in the titles and role scope of care navigators highlights increasing demand for system integration, however, the high variability of interchangeable terms and overlapping tasks create complexity for service users, providers, and researchers. An international Delphi study could create a consensus on the nomenclature and taxonomy for navigator roles that interface between health and social care systems. Developing professional identities, training, and strategies to seamlessly embed such roles into existing health and social care structures is also needed.

**Systematic Review Registration:**

https://www.crd.york.ac.uk/PROSPERO/, PROSPERO #CRD42024572605.

## Introduction

The breadth and fragmentation of health and social care systems creates a complex environment for individuals living with complex health and social care needs to navigate (1, 2). Recognising the benefits of integrating care for improving the outcomes of service users (3), a workforce of care navigators are being employed internationally to support individuals in navigating health and social care services. Understanding the various roles that exist in this workforce is critical to support effective implementation, positive service user and worker experiences, and to aid the development of robust professional identities for navigators that work between health and social care systems.

Care navigator roles may operate within a variety of established models of care, such as social prescribing, case management, and care coordination, with the main aim of such models being increased access to health and social care services, care integration and smoother patient journeys through health and social care services. Patient navigation originated as a model of cancer care in the United States (US), with the primary objective of patient navigators being to eliminate access barriers to healthcare and connect individuals with available services (4). Care coordination is similarly understood as an intervention through which care coordinators organise service user activities and referrals to optimise care delivery (5). This often involves information sharing and coordinating across multidisciplinary teams, with the model likened to concepts of case management (6). In this approach, care coordinators typically assess the needs and preferences of individuals, support care planning, and then manage and monitor the process of care itself (7). Conceptualised as distinct to care coordination (8), social prescribing was developed in the United Kingdom (UK) and involves link workers connecting individuals to non-clinical services and supports in their local community that address their wider social needs, such as those related to loneliness, housing instability, and other social determinants impacting on their health and wellbeing (9). Across the described models of care, the core function of the worker is consistent: to assist individuals in navigating needed services and supports. To ensure clarity in this umbrella review, the term “care navigator” is used to refer generally to any worker who navigates care between health, social, and community care systems. This “care navigator” terminology has been adopted in a UK competency framework to cover the range of aforementioned roles, though in practice, the framework also recognises that navigators work under various titles and in different models of care (10).

Effective care navigators can improve access to, and satisfaction with, health and social care services (11–13). However, there is a lack of understanding regarding the definition, structural organisation, and standardised role functions for this growing workforce, which prevents comprehending impact mechanisms in more depth. Conceptualisation of roles are often specific to the context of locally implemented programs, resulting in evaluations that are only relevant under a specific model of care, population, or geographical location. For example, literature on patient navigators is often focused on supporting individuals and families with specific health needs, such as cancer (4), dementia (14), and human immunodeficiency virus (HIV) (15), with models of care established in various local primary and tertiary health settings. By comparison, programs involving social prescribing link workers are usually implemented and evaluated in the context of primary care, typically targeting areas of socio-economic deprivation or to support individuals with “socially determined” long-term health and social needs (16–19).

Where navigation programs are grouped and studied under a conceptual model of care (e.g., social prescribing), there is also substantial variability in how the workforce is described and understood. Zurynski et al. (20) identified over 18 separate titles used to describe navigators within social prescribing initiatives, including link worker, care navigator, coordinator, social prescriber, and wellbeing coordinator; another review on social prescribing identified 75 different titles for the navigator roles in schemes implemented in the UK (21). The patient navigation field also contains marked variation in titles used, such as patient navigator, care coordinator, care manager, and system navigator (22, 23). This variability creates uncertainty for other health and social care workers about navigators' role scopes and boundaries (24, 25), creating challenges for the successful embedding of these new roles in health and social care systems and for a sense of professional identity for the worker. This may be especially challenging for roles that span the boundaries of health, social, and community care services, where successful implementation relies on suitable intersectoral understanding and collaboration (26). The limited understanding among service users and other providers about navigator functions may also create confusion, potentially leading to under-utilisation or over-dependence on the navigator and impacting the care of the service user (27).

Numerous reviews have synthesised knowledge about tasks, responsibilities, and organisational structures of care navigators, however, these are often limited to a specific program concept, such as patient navigation (28, 29) or social prescribing (30, 31). Moreover, the scope of previous reviews has been limited to national contexts, such as reviews focused on social prescribing in the UK only (27, 32), despite evidence that social prescribing has been implemented globally (9). This continued separation contributes to the poor understanding of the commonalities and differences in care navigation activities more broadly, and limits opportunities for the consolidation of role functions and terminologies in an international context. Counterintuitive to the purpose of integrated care, this lack of clarity on role titles and role functions may create confusion and further complexity. For example, service users and providers may misinterpret care navigator role functions, which could result in inappropriate referrals, such as the referral of service users with acute mental health needs to a general community signposting navigator program (24). Furthermore, limited understanding of the navigator role boundaries by a referring professional or service user may contribute to the navigator experiencing work overload or frustration (33), due to the navigator performing roles beyond their scope.

A clearer understanding of differences and similarities of role scopes, tasks and responsibilities across different navigator role titles and contexts is needed to support the effectiveness of navigation programs and the strengthening of professional identities. A clearly understood professional identity may provide greater recognition of how these roles contribute to achieving improved care experiences and outcomes for individuals. The overarching objective of this umbrella review project was to synthesise evidence on care navigator roles that support individuals who need to access to services provided in both health and social care systems. The current paper focuses on delineating and consolidating role titles, tasks, and responsibilities of care navigators.

## Methods

This umbrella review was prospectively registered on PROSPERO (CRD42024572605), and was informed by the Joanna Briggs Institute (JBI) umbrella review guidance (34) and is reported in accordance with the Preferred Reporting Items for Systematic Reviews and Meta-Analyses (PRISMA) guidelines (35).

### Search strategy and screening processes

The search strategy was developed in consultation with two medical librarians and included keywords and Medical Subject Headings (MeSH) terms related to the navigator role (e.g., “link worker”, “care navigator”, “care coordinator”), outcomes related to role experiences in practice (e.g., “role”, “scope of practice”, “educat*”), and article type (e.g., “review*”). MEDLINE (Ovid), Embase, PsycINFO, Cumulative Index to Nursing and Allied Health Literature (CINAHL), and Scopus were searched from 1 January 2019–31 May 2024 (Supplementary File S1). The Cochrane Database of Systematic Reviews was not searched, as preliminary searches did not identify any relevant articles. A five-year search period was chosen to allow for the most recent evidence to be summarised in this rapidly developing field. References were imported from each database into EndNote™ (X20). Duplicates were removed using SR Accelerator De-Duplicator software (36), and in Rayyan®, an online screening tool (37).

Review articles were eligible for inclusion if they were published in English, peer-reviewed, and utilised a systematic, scoping, or other rigorous review methodology [i.e., informed by a framework or reporting standards, such as PRISMA (35)]. Narrative reviews and umbrella reviews were excluded. Articles were included if they described workers whose roles required them to interface with healthcare and social care settings to assist individuals to navigate care that addressed both health and social care needs. The worker also had to be in a distinct role as care navigator; articles that described care navigation tasks being added on top of existing roles (e.g., general practitioner) were excluded. The worker could have any background or skills but were required to be employed in their role (i.e., non-voluntary, paid role) and to deliver person-centred care beyond simply providing education and/or emotional support to service users. Articles were included if they addressed at least one relevant element: role task, scope of practice, experiences and challenges, adaptations, training and education, qualifications, competencies, policies and guidelines. This paper focuses only on synthesising elements of role tasks, scope of practice, and role titles, where identified. Findings about the elements not discussed in this paper will be reported elsewhere. Articles that reported on the effectiveness or other outcomes of care navigation programs without explicit detail of the navigator role were excluded.

Title and abstract screening was conducted using Rayyan®, an online screening tool (37), by three reviewers working as a team (SW, YZ, LW), using dual independent screening. Conflicts were resolved through discussion at two meetings. Full-text screening was conducted in a custom-built Microsoft Excel workbook. For full-text screening, 10% of the articles were screened independently by a team of eight reviewers (SW, YZ, LW, CR, AS, ZT, SL, NG) to ensure consistency. The results of this 10% screening were discussed between all authors, and discrepancies resolved. The remaining 90% of articles were screened independently in pairs between the same eight reviewers. There was 73% agreement between reviewers; conflicts were resolved by two arbiters (SW, YZ) or discussed with the review team in regular meetings. Additional articles were also identified from a search within a scoping review conducted by the author team (38), and were subject to the same screening processes.

### Data extraction and synthesis

One included study was independently extracted by seven reviewers (SW, YZ, LW, AS, ZT, SL, NG) to ensure consistency and to pilot the extraction form. The data extraction form was revised based on discussion among four reviewers (SW, YZ, ZT, NG). The remaining articles were split between the team of reviewers (SW, YZ, LW, AS, ZT, SL, NG, CR), with each article independently extracted by one reviewer. Data extraction fields included: publication details (country of first author; aims; review type), review methodology (database searches; inclusion criteria; methods, year, and country of included studies; quality assessment and synthesis details), description of the care navigator (title, health setting, population served, social setting), and key elements (tasks; scope; mode and frequency of contact; role experiences and challenges; education, qualifications, professional development; competencies and competency frameworks; policies and guidelines). Only roles that discussed navigation between health and social care settings were extracted (i.e., navigation within the health system only was excluded). Barriers and enablers relevant to the role, identified needs, and proposed solutions were also extracted from each included article. Two reviewers (SW, LW) verified the data for accuracy prior to synthesis, by cross-checking extracted data against included articles.

Data analysis was conducted in Microsoft Excel using a narrative synthesis approach (39, 40). For the narrative synthesis, one author (SW) first familiarised themselves with all data, then conducted all analyses. Characteristics of the included reviews (e.g., review methodology, country of included sources) were categorised inductively by grouping data based on observed shared content. These characteristics were tabulated in Microsoft Excel and described using frequencies. Role titles in primary sources were included in analysis if they were reported in the results (or the included studies' Supplementary materials). Role titles were analysed and grouped into categories; role categories were used to stratify reporting of other elements where relevant. Elements related to role scope and task domains were synthesised across included reviews by one author (SW) using an inductive approach. Initial themes related to role scope and task domains were discussed within a small group (SW, CR, YZ, LW), who provided feedback and amended the theme descriptions. One author (SW) then revised these themes with verification provided by the whole author team.

### Overlap of evidence

To assess the overlap of evidence within included reviews, one author (LW) imported the primary sources included in each review into EndNote™ (X20). Primary sources were typically identified using tables that described the characteristics of each of the included studies' (supplementary materials), or through citations in results sections of the included reviews. These references were verified by another author (SW) to ensure accuracy. The overlap of evidence, specifically the inclusion of the same primary source in multiple included reviews, was determined by one author (SW) checking for duplicates (using EndNote™ and manually) amongst identified primary sources.

### Quality appraisal

Quality appraisal of included reviews was conducted for most using the JBI Checklist for Systematic Reviews (34). The JBI Checklist for Systematic Reviews assesses whether a systematic review contained appropriate elements, such as appropriate inclusion criteria, search strategy, and processes for data extraction and synthesis (34). For realist reviews, the Realist And Meta-narrative Evidence Syntheses: Evolving Standards (RAMESES) Quality Standards for Realist Syntheses and Meta-Narrative Reviews was used (41). The RAMESES Quality Standards guide researchers to appraise the adequacy of elements, such as the sufficient focus of the review, the application of realist philosophy and logic, and the construction of realist programme theory (41). Reviews were considered high quality if they were appraised with all “Yes” responses using the JBI Checklist, or had all aspects appraised with “Adequate” or higher for the RAMESES Quality Standards. Five included articles were independently screened by four reviewers (YZ, SW, CR, LW); conflicts were resolved through consensus. The remaining articles were allocated among the same four reviewers and assessed independently, and any discrepancies were resolved through discussion amongst the four reviewers, and cross-checking against the quality assessment tool.

## Results

### Study selection

**Figure 1:**
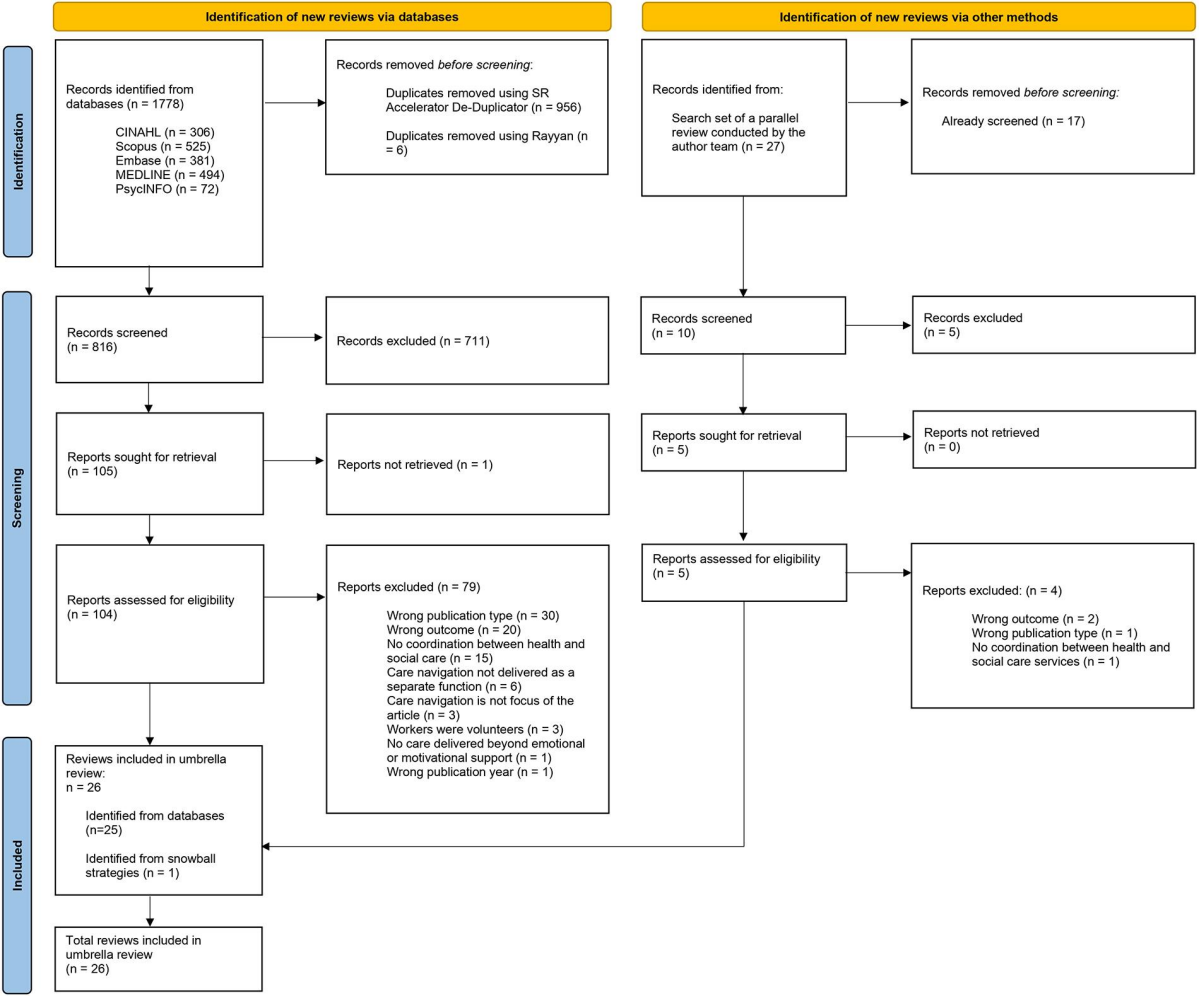
PRISMA flow diagram.

Database searches identified 1778 review articles for screening; 962 of these were duplicates, leaving 816 for title and abstract screening. One hundred and five articles met inclusion criteria for full-text screening. Five additional review articles were identified for full-text screening from a library developed as part of a separate review conducted by the research team. A total of 26 review articles were included (Figure 1).

### Characteristics of included reviews

The included 26 reviews are described in Table 1. Seventeen (65%) were published between 2022 and 2024 and over half (*n* = 15, 58%) were led by authors from Canada (*n* = 6), the US (*n* = 5), and the United Kingdom (UK; *n* = 4) (Table 1). Included reviews mostly applied either scoping (*n* = 14) or systematic (*n* = 8) review methodology. Two reviews used realist review methodology (42, 43), one review was an integrative review (30), and another review used a structured literature review approach following various reporting guidelines (44).

**Table 1:** Characteristics of included reviews.

First author	Objectives	Sources of included articles (search dates)	Characteristics of included sources	Service user population and context	Health settings
Systematic reviews					
Desveaux et al. (45)	To generate a preliminary program theory to describe how patient navigator roles are designed and delivered; and describe how the resulting program theory was applied in context to inform a prototype navigator program	Secondary analysis of published systematic review; MEDLINE, Embase, CENTRAL, PsycINFO, Social Work Abstracts (inception to 23 August 2017)	*N* = 21 included studies.	Variable populations and context. Focused populations include patient navigation programs for diabetes (*n* = 7), cancer (*n* = 3), HIV (*n* = 6), kidney disease (*n* = 2), and multiple chronic conditions (*n* = 1)	Primary care
			Trials with control and intervention groups.		
			Countries not reported.		
Ebrahimoghli et al. (46)	To identify and categorise factors influencing social prescribing initiatives	PubMed, Scopus, ISI Web of Knowledge (date range not reported)	*N* = 22 included studies.	Not reported	Primary care
			Qualitative studies.		
			Studies conducted in the United Kingdom (UK) (*n* = 19), the Netherlands (*n* = 1), Spain (*n* = 1), Norway (*n* = 1).		
Harris et al. (23)	To systematically review the published literature on patient navigation programs addressing the mental health of parents and caregivers during the perinatal period, including existing models, effectiveness, patient and provider perceptions, and facilitators and barriers to program success	CINAHL, Cochrane Library, Maternity & Infant Care (Ovid SP), Embase, PsycINFO, PubMed (January 1990–November 2021)	*N* = 19 included studies.	“Women or families identified as being at high risk of mental health disorders during the perinatal and early childhood period, and less likely to receive adequate care”. Diverse setting, including families from low-income or ethnic minority backgrounds	Family care settings (e.g., family and paediatric medical clinics, obstetrics and gynaecology practices, childcare centres); primary care
			Mixed-methods pre-post designs.		
			All studies conducted in the US (*n* = 19).		
Koenig et al. (54)	To identify and characterize the activities of HIV patient navigators; to determine which were most common; whether there were essential or defining features; and whether distinct elements of navigator interventions could be distinguished	MEDLINE (Ovid), Embase (Ovid), PsycINFO (Ovid), and CINAHL (EBSCOhost); supplementary searches in PubMed, Scopus, New York Academy of Medicine literature database, citation tracking (searched 1 January 1996–23 April 2018)	*N* = 24 included studies.	Persons with HIV, including those from ethnic, social, and racial minority backgrounds and/or other disadvantaged circumstances.	Primary care clinics, general practice (GP) offices
			Included studies used the following methods: qualitative (*n* = 23), commentaries (*n* = 12), reviews/guidelines (*n* = 12), protocols (*n* = 5), linked studies (*n* = 4), other (*n* = 12).		
			All studies conducted in the United States (US) (*n* = 24).		
Roland et al. (56)	To understand and describe client experiences with HIV patient navigation, in order to facilitate research to practice for the benefit of public health programs and practitioners	MEDLINE (Ovid), Embase (Ovid), PsycINFO (Ovid), CINAHL (EBSCOhost) (1 January 1996–15 October 2018)	*N* = 7 included studies.	Persons with HIV, including those who are of low socio-economic or culturally and linguistically diverse backgrounds, were previously incarcerated, or part of the LGBTQIA + community	Community health
			Methods included intervention studies (*n* = 6), and ethnographic studies (*n* = 1).		
			All studies conducted in the US (*n* = 7).		
Roland et al. (57)	To advance public health research and practice by articulating key experiences and perspectives of HIV patient navigators	MEDLINE (Ovid), Embase (Ovid), PsycINFO (Ovid), CINAHL (EBSCOhost) (1 January 1996–19 October 2020)	*N* = 9 included studies.	Persons with HIV, including those who are of low socio-economic or culturally and linguistically diverse backgrounds, were previously incarcerated, or part of the LGBTQIA + community	Community health
			Methods included qualitative studies (*n* = 9).		
			All studies conducted in the US (*n* = 9).		
Rapo et al. (50)	To identify and evaluate critical components within social prescribing programmes that can impact loneliness, health, or well-being among older adults	PubMed, MEDLINE, PsycINFO, CINAHL, SOCIndex (1 March 2020)	*N* = 17 included articles.	Patients with some form of long-term condition, poor physical or mental health, frequent healthcare visits, or having experienced a recent life event such as loss of spouse, unemployment, or retirement. Settings varied: urban (*n* = 3), rural (*n* = 1), socioeconomically deprived (*n* = 4)	Primary care
			Methods of articles included systematic reviews (*n* = 4), longitudinal studies (*n* = 2), qualitative studies (*n* = 3), mixed-methods studies (*n* = 2).		
			Country of authorship reported as UK (*n* = 11), and the Netherlands (*n* = 1). Most contexts were in the UK.		
Zhang et al. (27)	To examine the social prescribing approaches that have been evaluated for migrants, their effects on migrants, and the experiences of implementation from the perspectives of migrants, providers and referrers to social prescribing programmes	Embase, PsycINFO, Web of Science, Social Policy & Practice (1 January 2000–2 June 2020). Grey literature included	*N* = 32 included articles.	Focused on international migrants	Primary care, GPs, community-based organisations
			Methods included: programme evaluations (*n* = 12), case reports (*n* = 11), qualitative studies (*n* = 7), stakeholder consultation event summaries (*n* = 2).		
			All studies published in the UK (other countries excluded).		
Scoping reviews					
Choi et al. (52)	To identify interprofessional competencies for professionals who provide integrated community care for older adults	PubMed, CINAHL, Embase, Cochrane Library, internet sources (1990–2020)	*N* = 10 included studies.	Older adults	Community care
			Methods not reported.		
			Countries of included studies were England (*n* = 5), US (*n* = 3), Finland (*n* = 2).		
Doucet et al. (29)	To map the literature on the characteristics, impact, barriers and facilitators of hospital-based patient navigation programmes that support patients who experience injury-related trauma and their caregivers	CINAHL (EBSCOhost), Embase (Elsevier), ProQuest Nursing & Allied Health (ProQuest), PsycINFO (EBSCOhost), and MEDLINE and Epub Ahead of Print, In-Process, In-Data-Review & Other Non-Indexed Citations, Daily and Versions, ProQuest, Google Scholar, websites (search conducted 6 June 2021; grey literature searched between September and October 2021)	*N* = 11 included studies.	Hospital patients who experienced injury-related trauma and their caregivers after discharge into the community	Hospital
			Methods included descriptive studies (*n* = 3), pilot prospective cohort designs (*n* = 1), case study (*n* = 1), unpublished grey literature (*n* = 6).		
			All studies conducted in the US (*n* = 11).		
Kelly et al. (47)	To delineate the differences in the functions and backgrounds of patient navigators and case managers	Medline, CINAHL, PubMed, Google (search last conducted June 2018)	*N* = 160 included studies.	Varied between studies; *n* = 51 studies focused on cancer care	Hospital, community health
			Methods not reported.		
			Countries of included studies were the US (*n* = 120), Canada (*n* = 26), UK (*n* = 5), Sweden (*n* = 3), Australia (*n* = 3), France (*n* = 1), Pakistan (*n* = 1), Singapore (*n* = 1).		
Kokorelias et al. (22)	1. Identify and describe the literature on patient navigator programs that employ occupational therapists, including the roles and functions of patient navigators as well as the settings and populations they serve 2. Map the identified functions of patient navigators onto the 2021 Competencies for Occupational Therapists in Canada	PsycINFO, OT Seeker, MEDLINE (Ovid), Embase, OT Critically Appraised Topics (CATs), CINAHL, Campbell Collaboration, Agency for Health Care Policy and Research, American Occupational Therapy Association (AOTA) Evidence Briefs, and Canadian OT Foundation Critical Research Literature Reviews, Google (search last conducted 8 August 2022)	*N* = 10 included studies.	Varied between studies, including homeless people (*n* = 1), patients with stroke (*n* = 3), gender diverse adults (*n* = 2), adults with spinal cord injury (*n* = 2), all patients (*n* = 1), and cancer patients (*n* = 2)	Community health, hospitals
			Methods of included studies were letter to the editor/commentaries (*n* = 3), mixed-methods (*n* = 1), qualitative (*n* = 1), quantitative (*n* = 1), not known (*n* = 4).		
			Countries of included studies were the US (*n* = 3), Canada (*n* = 5), and UK (*n* = 2).		
Mullen et al. (48)	To describe the origins of patient navigation and its relevance to the field of mental health and/or addictions	MEDLINE, PsycINFO, AMED, Embase, CINAHL (searched on 1 August 2019)	*N* = 26 included studies.	Varied, but included patients with serious mental illness (*n* = 5), patients with both physical and mental health concerns (*n* = 4), patients with criminal justice issues (*n* = 2), youth and families (*n* = 1)	Community health
			Methods of included studies were randomised controlled trials (RCTs) (*n* = 7), case descriptions (*n* = 5), preliminary evaluations (*n* = 5), pilot studies (*n* = 3), Delphi studies (*n* = 1), mixed-methods studies (*n* = 1).		
			Countries of included studies were US (77%), Canada (19%), and UK (4%).		
O’Grady et al. (49)	To identify and describe the available international evidence regarding processes of referral to an intermediary, the characteristics of referred community-dwelling adults, and the processes and outcomes of connecting referred individuals to local physical activity and exercise	Embase, MEDLINE, Web of Science, and CINAHL, unspecified grey literature (inception to June 2022)	*N* = 28 included studies.	No target population	Non-specified clinical settings, community and voluntary sectors
			Methods included quantitative studies (*n* = 14), qualitative studies (*n* = 7), mixed and other method studies (*n* = 14).		
			Countries of included studies were England (*n* = 5), Scotland (*n* = 4), Wales (*n* = 1), USA (*n* = 9), Canada (*n* = 2), the Netherlands (*n* = 2).		
Österholm et al. (58)	To map how different health and care agencies collaborate and coordinate for older people with dementia	PubMed; CINAHL; Embase; PsycINFO; Scopus, Web of Science (January 2012–6 April 2022)	*N* = 56 included studies.	Focused on older people with dementia, their carers	Community health
			Methods include qualitative studies (*n* = 24), quantitative or RCT studies (*n* = 20), mixed-methods studies (*n* = 12).		
			Geographic regions of included studies were Europe (*n* = 25), North America (*n* = 22), Asia (*n* = 4), Oceania (*n* = 3), Asia and Europe (*n* = 1), Oceania, North America, Europe (*n* = 1).		
Rankin et al. (55)	1. To identify the extent and the nature of research pertaining to the role of the indigenous patient navigator in Canada, the US, Australia, and New Zealand 2. Examine barriers faced by indigenous peoples when utilising Western Health services 3. Identify potential gaps in the existing published literature and key research priorities, which will assist to inform indigenous patient navigator role development and practice as well as advance related health policies	Medline, CINAHL, Web of Science, iPortal.usask.ca (1990–August 2019)	*N* = 16 included studies.	Indigenous patients, living within indigenous communities	Cancer care, primary care, community health, home care, tertiary care/hospital
			Methods of included studies are qualitative (*n* = 7), quantitative (*n* = 6) mixed-method convergent design (*n* = 1).		
			Countries of included studies were the US (*n* = 12), Canada (*n* = 3), New Zealand (*n* = 1).		
Richard et al. (59)	To analyse the conditions under which health mediation for healthcare use is successful and feasible when applied to underserved populations and those exposed to numerous vulnerabilities, such as people living in precarious habitats, travellers, migrants and homeless people	Cairn, PubMed, Scopus, PsycINFO (1 January 2015–18 December 2020)	*N* = 22 included studies.	Focus generally on underserved or vulnerable populations	Community health
			Methods of included studies were case studies (*n* = 12), literature reviews (*n* = 7), cohort studies (*n* = 2), RCTs (*n* = 1).		
			Countries of included studies were US (*n* = 11), France (*n* = 9), UK (*n* = 1), Australia (*n* = 1).		
Sandhu et al. (31)	To identify and categorise the components of link worker social prescribing schemes in the United Kingdom	MEDLINE, Embase, CINAHL, PsycINFO, Web of Science, Scopus (January 2000–June 2021)	*N* = 33 included studies.	Patients living in disadvantaged regions or areas with high levels of socio-economic deprivation. Patient characteristics varied	Community health, primary care
			Methods of included studies were RCT (*n* = 1), quasi-experimental cluster RCT (*n* = 1), uncontrolled before and after study (*n* = 6), mixed-methods (*n* = 4), qualitative studies (*n* = 16) case studies (*n* = 4).		
			Countries of included studies were England (*n* = 19), Scotland (*n* = 2), Ireland (*n* = 1).		
Shockney et al. (60)	To identify trends in navigation practice within the chronic disease space	PubMed, MEDLINE (1 January 2010–11 September 2020)	*N* = 33 included studies.	Chronic diseases	Hospital, primary care, home care, other specialised clinics
			Mixed methods studies.		
			Studies from US, UK, Germany (otherwise not reported).		
Stretton et al. (53)	To scope and map the landscape of case management work in New Zealand, specifically investigating the jobs, roles, and relationships of case managers in Aotearoa New Zealand	CINAHL (EBSCO), Medline (EBSCO), ERIC (Ovid), Scopus, and Google Scholar; open internet searches (2000–March 2022)	*N* = 148 included sources.	Populations varied, including patients with long-term conditions, and specific cultural populations	Primary care, community health
			Methods of included sources were peer-reviewed articles (*n* = 35), book chapters (*n* = 2), theses (*n* = 6) reports and evaluations (*n* = 28).		
			All sources based in New Zealand (*n* = 148)		
Surugiu et al. (51)	To unveil opportunities for social prescribing and its value in unburdening healthcare and offering sustainability	PubMed, MEDLINE (dates not specified)	Number of studies not reported.	No target population	Primary care
			Qualitative and quantitative methods.		
			Countries of included studies not reported.		
Yadav et al. (62)	To explore the existing social prescribing programs for people with long-term chronic conditions and identify the opportunities and challenges of implementing such initiatives in primary health care settings	MEDLINE via PubMed, Embase, Web of Science (January 2010–June 2023)	*N* = 15 included studies.	Patients with long-term chronic conditions. Contexts include areas of socio-economic deprivation (*n* = 6) and culturally and linguistically diverse backgrounds	Primary care
			Methods of included studies were cohort study (*n* = 1), cross-sectional study (*n* = 1), pragmatic study (*n* = 1), mixed-methods study (*n* = 4), qualitative study (*n* = 8).		
			Countries of included studies were UK (*n* = 14), Australia (*n* = 1).		
Other reviews					
Calderon-Larranga et al. (42)	To define best practice social prescribing by identifying context-specific enablers and tensions, and from these to build a comprehensive framework which could be used for theorising and evaluating social prescribing in primary care	Medline (Ovid), Embase (Ovid), PsycINFO (Ovid), Scopus (Elsevier), CINAHL Plus (EBSCO), PubMed (NCBI), Web of Science (Clarivate analytics), International Bibliography of the Social Sciences (IBSS) (ProQuest), The Cochrane Library, OpenGrey (INISR-CNRS), Campbell Collaboration, The King's Fund, LILACS (BIREME) (searched 1 September 2019)	*N* = 140 included sources.	No target population	Primary care
			Methods of included sources were qualitative (*n* = 40), quantitative (*n* = 37), mixed-methods (*n* = 36), literature reviews (*n* = 25), research-based toolkit (*n* = 1), evaluability assessment study (*n* = 1).		
			Countries not reported in main results.		
Linceviciute et al. (30)	To identify the existing ways in which link workers might support the needs of those living with multiple long-term conditions	PubMed, Web of Science, Social Care Online (SCIE), CINAHL, PsycINFO, Social Prescribing Network, National Academy for Social Prescribing, Oxford Social Prescribing Research Network, Google Scholar (searches conducted 2017–May 2023)	*N* = 18 included studies.	Adults aged 40–74, with multiple long-term conditions	Primary care, community health
			Methods of included studies were qualitative (*n* = 10), observational (*n* = 3), literature review (*n* = 3), experimental (*n* = 2).		
			Countries of included studies were UK (*n* = 15), Ireland (*n* = 1), unspecified (*n* = 2).		
Rothe et al. (44)	To examine the role of the link workers, activities, and target groups involved in social prescribing	PubMed, PsycINFO, CINAHL, Web of Science, Cochrane Library (studies published before May 2020)	*N* = 16 included studies.	Patients with psychosocial and mental health issues, and patients with physical health issues	Not reported
			Methods of included studies were quantitative (*n* = 6), RCTs (*n* = 2), qualitative (*n* = 7), mixed-methods (*n* = 3).		
			Countries of included studies were England (*n* = 11), Scotland (*n* = 1), Australia (*n* = 1), the Netherlands (*n* = 1), not reported (*n* = 2).		
Tierney et al. (43)	1. What are the outcomes associated with social prescribing connector schemes in primary care? 2. What are the mechanisms that produce these outcomes? 3. Under what conditions (context) are these mechanisms activated?	ASSIA, CINAHL, Cochrane Database of Systematic Reviews, Cochrane Central Register of Controlled Trials, DARE, Dissertations and Abstracts, Embase, HMIC, MEDLINE, SCI and SSCI, NHS policy documents, non-published evaluations from clinical commissioning groups, other documents. (Sources searched May 2018)	*N* = 118 included sources.	No target population	Primary care
			Methods of included sources were quantitative (*n* = 25), qualitative (*n* = 16), mixed-methods (*n* = 57), descriptive report/commentary (*n* = 12), RCT (*n* = 2), review (*n* = 3), grey literature (*n* = 3).		

Twelve (46%) of the included reviews either searched from database inception or did not use a specific start date for their search (22, 29, 42–51). In addition to peer-reviewed literature, 11 reviews searched grey literature for relevant navigation program resources (22, 27, 29, 30, 42, 43, 47, 49, 51–53). Two reviews specified that un-published evaluations of social prescribing programs were requested from UK government agencies (27, 43). Most reviews typically included studies from high-income countries, such as the UK, the US, Australia, Canada, the Netherlands, and New Zealand; eight reviews restricted the inclusion of primary studies based on country (27, 31, 43, 53–57). Across the reviews that reported the country of primary sources, only two studies were included from low to middle income countries (i.e., Pakistan and Thailand) (47, 58).

### Overlap of evidence

A total of 1,021 primary sources were synthesised within the 26 included reviews, including peer-reviewed literature, and grey literature such as non-peer-reviewed program evaluations. The number of sources (i.e., journal articles and/or grey literature) in included reviews ranged from 10 (22) to 160 (47). Among all included primary sources, 957 were referenced in sufficient detail to be assessed for overlap of primary evidence. Of these 957 primary sources, 133 were referenced across multiple reviews, representing a 14% overlap of primary evidence. Most of these overlapping primary sources were reports or evaluations of social prescribing schemes. Therefore, 824 unique sources informed the reviews included under this umbrella review.

### Quality of included reviews

Twenty-four (92%) of the included reviews were appraised using the JBI Checklist for Systematic Reviews (34) and eight (33%) were of high quality (Supplementary Table 1). Of the reviews not deemed high quality, nine included limited descriptions of methods to minimise errors in data extraction, or did not report this at all (23, 44, 47, 50, 51, 53, 55, 59, 60), and five reviews (23, 44, 47, 51, 60) did not clearly describe the methods used to combine study outcomes. No reviews assessed the likelihood of publication bias, as no reviews conducted quantitative meta-analysis of primary studies. The two realist reviews included (42, 43) were appraised as high quality (Supplementary Table 2). One article was excluded during quality appraisal because it lacked sufficient detail regarding review methodology (61).

### Care navigation titles and role categories

Titles of the navigation workers varied substantially at the primary source level and at included review level. Given that each primary source was typically associated with a specific program or model, specific titles used for workers varied based on context and purpose. When synthesised within included reviews, titles from primary sources were usually consolidated and reported under a single role category. For example, Harris et al. (23) combined primary sources that used navigator, perinatal wellness navigator, maternity care coordinator, wellness navigator, care manager, patient navigator, and community health worker, into the role category of *Patient Navigator*. This approach was common across most included reviews (*n* = 23, 88%), and simplified reporting of outcomes. Reviews reporting on *Patient Navigators* as a role category also contained some variation for navigation programs designed for specific populations, such as HIV patient navigators (56, 57) and Indigenous patient navigators (55).

In included reviews that focused on a single role (*n* = 23), titles from primary sources were reported under the following role categories: *Patient Navigators* (*n* = 9) (22, 23, 29, 45, 48, 54–57), *Link Workers* (*n* = 9) (27, 30, 31, 42–44, 46, 50, 51), *Care Coordinators* (*n* = 2) (52, 58), *Intermediaries* (*n* = 1) (49), *Health Mediators* (*n* = 1) (59), and *Social Prescribers* (*n* = 1) (62). Within each role category, there was substantial variation of included titles from the primary sources of included reviews in this umbrella review. For instance, within reviews focused on *Link Workers*, 22 unique role titles were indicated for this category; for example, Sandhu et al. (31) included community link practitioner, community connector, wellbeing coordinator, and social prescribing coordinator under the broad role of *Link Worker*.

**Figure 2:**
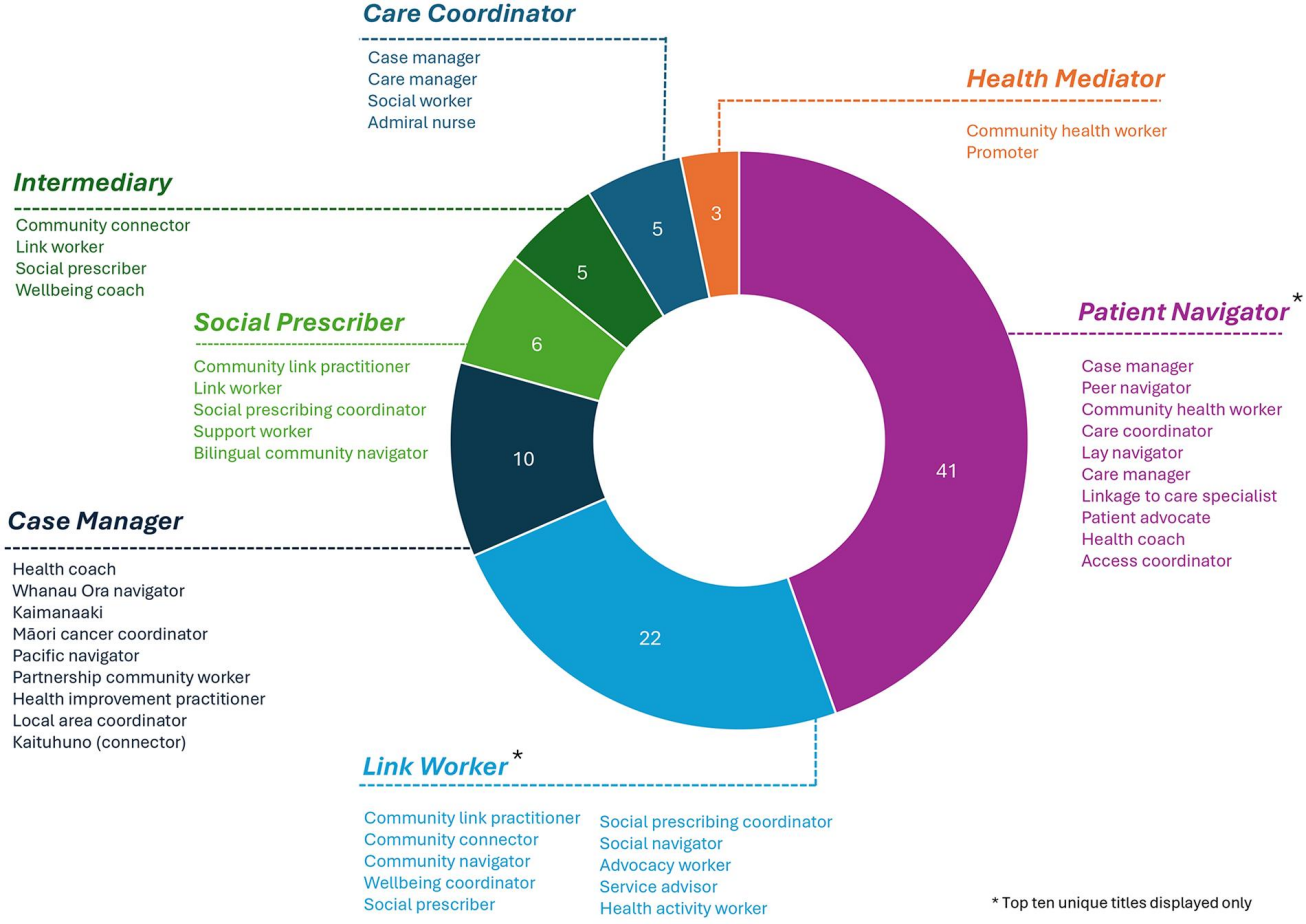
Care navigator role titles across role categories as reported in the included reviews. Role titles (below each dashed line) indicate terminology used in each primary source. Role categories (above dashed line; capitalised, bolded, and italicised) are how the titles were grouped in included reviews. Numerical data labels indicate the number of unique role titles reported under each role category. Role titles that are the same as the role category are included in the count but have not been listed.

Three reviews took a different approach to consolidating titles and roles of the navigation workforce (47, 53, 60). One review specifically delineated *Patient Navigators* separately to *Case Managers*, indicating the functional differences between their roles in health and social care settings (47). As part of a broad overview of navigation programs, nurse navigators, patient navigators, and care coordinators were described separately by Shockney et al. (60). In our analysis, nurse navigator and patient navigator were considered within the *Patient Navigator* category, and *Care Coordinator* as its own category because they tend to be described separately in other literature. Stretton et al. (53) reported on case management titles used in New Zealand including health and social navigators (Whanau Ora navigators, Kaimanaaki, Maori cancer coordinators, pacific navigators, partnership community workers), primary health care roles such as Health Improvement Practitioners and health coaches, and navigators that work with older adults and people with disability [local area coordinator, kaituhuno (connector)]. These roles were consolidated under the overarching role category of *Case Manager* (53). In our analysis, 78 unique titles were used across the 26 included reviews. The authors of the included reviews grouped these 78 role titles under seven broad categories (Figure 2): *Patient Navigator* (*n* = 11 reviews), *Link Worker* (*n* = 9 reviews), *Care Coordinator* (*n* = 3 reviews), *Case Manager* (*n* = 2 reviews), *Social Prescriber* (*n* = 1 review), *Intermediary* (*n* = 1 review), *Health Mediator* (*n* = 1 review).

#### Crossover and clustering of role titles among role categories

**Figure 3:**
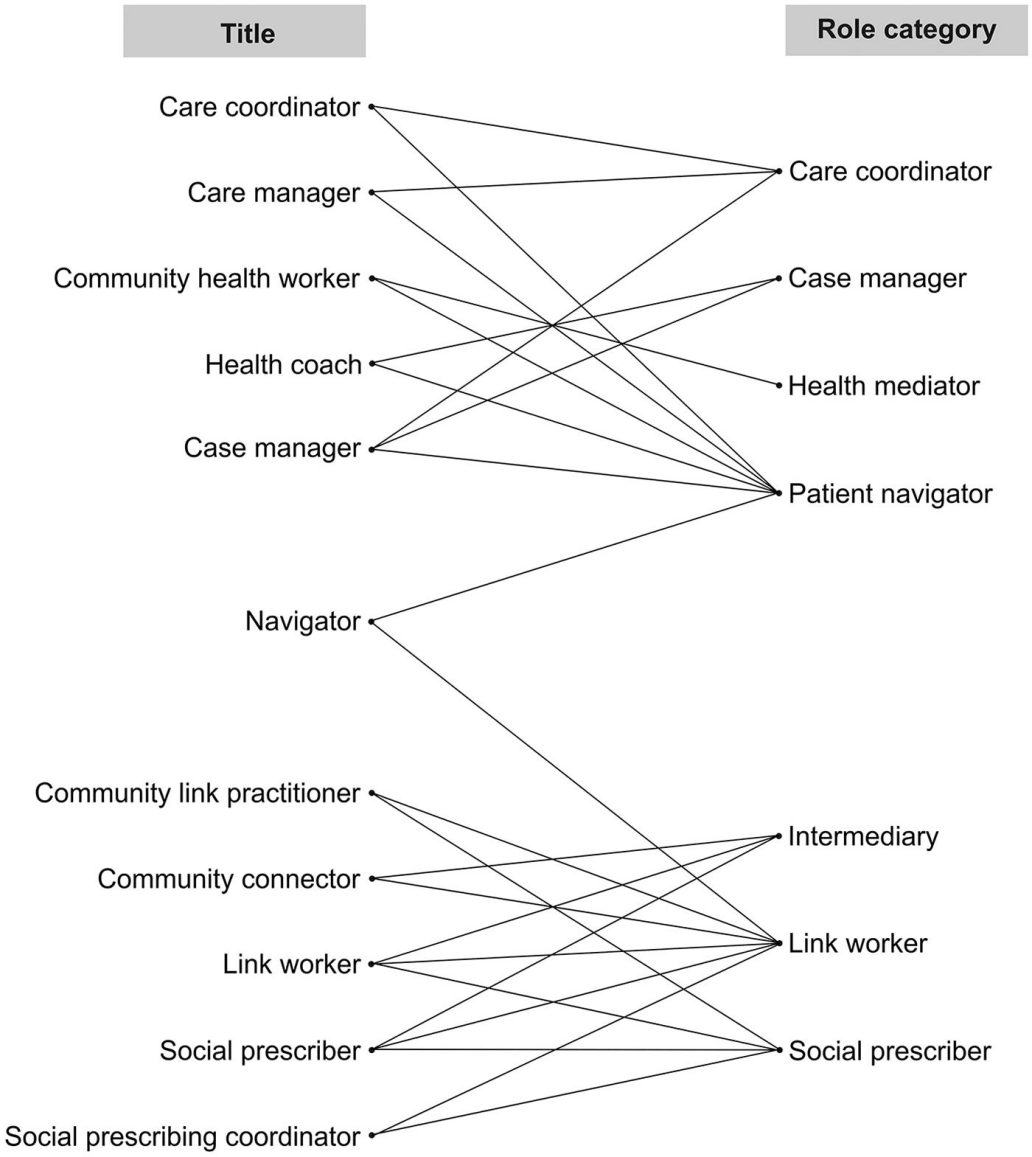
Crossover and clustering of role titles among role categories. Title refers to the terms used to describe care navigators in primary sources. Role category refers to the grouping of role within each included review, for example, titles of social prescriber and link worker grouped into the overall role category of Link Worker. Only titles that were present across multiple role categories included (*n* = 11 titles). Lines indicate links between titles and role categories.

Of the 78 unique role titles, 11 (14%) were used to describe workers across multiple categories; amongst these titles, two clusters of role category emerged (Figure 3). The categories of *Care Coordinator*, *Case Manager*, *Health Mediator* and *Patient Navigator* clustered around specific and often shared roles titles, including care manager, case manager, community health worker, and health coach. A second distinct cluster of *Link Worker*, *Social Prescriber* and *Intermediary* included the shared titles of link worker, community link practitioner, and social prescribing coordinator. The role title of navigator was present in both clusters, used for the role categories of *Patient Navigator* and *Link Worker*. This indicates that it may be used as a general title amongst the care navigation workforce.

### Health settings for the navigator workforce

Ten reviews focused specifically on *Link Workers* (*n* = 9) (27, 30, 31, 42–44, 46, 50, 51) or *Social Prescribers* (*n* = 1) (62) working in social prescribing programs. These programs were all implemented in primary or community health care settings.

Of the remaining 16 reviews, ten described navigation programs that were implemented in primary or community health care settings only (23, 45, 48, 49, 53, 54, 56–59). These programs were delivered by *Patient Navigators* (*n* = 6) (23, 45, 48, 54, 56, 57), *Care Coordinators* (*n* = 1) (58), *Health Mediators* (*n* = 1) (59), *Case Managers* (*n* = 1) (53), and *Intermediaries* (*n* = 1) (49).

Four reviews reported on navigation programs that were implemented in both primary or community health care settings, and hospital settings (22, 47, 55, 60). All four reviews included *Patient Navigators* as the primary role category. *Case Managers* and *Care Coordinators* were also described to work across primary or community, and hospital settings (47, 60). Only one review focused specifically on hospital-based navigation programs (29), which were delivered by *Patient Navigators*.

### Target populations of navigation programs

Fourteen reviews (54%) targeted navigation programs for specific groups (23, 27, 29, 30, 45, 48, 52, 54–58, 60, 62). Programs for older people (*n* = 3) (30, 52, 58), people with chronic or long-term physical health conditions (*n* = 4) (45, 48, 60, 62), and people with psychosocial or mental health problems (*n* = 2) (23, 48) were delivered by *Patient Navigators*, *Care Coordinators*, and *Link Workers*. Three reviews focused on people living with HIV (54, 56, 57), and were all delivered by *Patient Navigators*. Another review focused on *Patient Navigators* for individuals and caregivers after experiencing injury-related trauma (29). Two reviews included navigation programs for specific cultural groups: one review was focused on social prescribing for international migrants, delivered by *Link Workers* (27); another review focused on care navigation within Indigenous communities in Canada, US, Australia, and New Zealand, delivered by *Patient Navigators* (55).

### Service mode and frequency

Sixteen reviews (62%) reported the mode, frequency, and intensity with which the navigator worked with the service user (22, 23, 27, 30, 31, 43, 44, 47, 49, 50, 53, 56–58, 60, 62). Where reported, the mode of contact varied and was often determined based on the need and preference of the service user. Twelve reviews indicated the use of digital technologies, such as phone calls, text messages, emails, and videoconferencing for maintaining contact between navigators and service users (22, 23, 30, 31, 44, 47, 49, 50, 56, 58, 60, 62). Digital technologies were typically reported alongside face-to-face contact, however, two reviews reported on programs that were conducted exclusively over the phone (23, 60). One review specifically emphasised that the use of digital technologies, including connecting with service users on social media, allowed social prescribers to offer ongoing emotional support during the COVID-19 pandemic, when face-to-face contact was not possible (62).

Eight reviews reported on the duration of the program or number of contacts between navigator and service users (23, 30, 31, 44, 47, 49, 50, 56). Details varied substantially among reviews, however, service users generally engaged with programs within a 6-month period, with contact modality and frequency jointly determined by the service user needs or preference and navigator discretion. The longest program duration was five years (50). Three reviews specified that contact is the most intense at the beginning of the navigation program (31, 44, 49), either through longer appointments or increased frequency of contact. Some navigation programs had a fixed number of sessions (23, 44, 50), with two reviews of social prescribing programs highlighting that this was to reduce service user dependency on the service (44, 50). One review indicated that for case managers in New Zealand, no follow-up sessions were scheduled after the initial appointment, placing the onus on the service user to initiate engagement with the service (53). Feedback related to the frequency of service provision was reported in one review, with some stakeholders (service users, providers, referrers) noting they felt sessions were too infrequent or short, while others valued the flexibility (27). There were no observable differences in service mode and frequency between role categories.

### Professional interactions and task domains

Navigators interacted with a variety of health, community, and social care providers. This included linking service users with community activities (e.g., gardening clubs, arts and crafts activities), physical activity and healthy lifestyle programs, welfare programs and other social services (e.g., financial support, legal and housing services, food banks), and physical and psychological health services where needed.

Twenty-three reviews (88%) reported sufficient detail on care navigator tasks and responsibilities performed by navigators across many different categories and models of care. Six task domains emerged from the synthesis and are described in Table 2. The most frequently identified task domain was navigation (*n* = 22 reviews, 96%), which included the core function of helping service users navigate services between health and social care systems. Capacity building and supporting self-management was also frequently reported (*n* = 20 reviews, 87%), and included the provision of education, emotional support, and goal setting to service users. The care navigator was also responsible for building relationships with service users and delivering personalised care (*n* = 18 reviews, 78%); an assessment of health and wellbeing needs and the development of action plans was commonly reported in this domain. Assisting service users with practical needs was reported in 17 reviews (74%), and included accompanying service users to activities, assisting with paperwork and transportation arrangements, or acting as language interpreters. Non-service-user facing tasks (*n* = 15 reviews, 65%), such as collaborating with community and health organisations, raising awareness of the service, and managing client lists were also described. Six reviews (26%) identified that care navigators with appropriate training or qualifications performed clinical tasks secondary to the core navigation function, such as conducting physical health assessments and being involved in clinical treatment delivery. Another five reviews (22%) reported that the professional background of navigators (e.g., nursing, occupational therapist) may have broadened the scope of their navigation role to include clinical aspects.

**Table 2:** Task domains identified across the included reviews.

Task Domain	Description	Role Categories	References
Navigation (*n* = 22 reviews)	Helping service users navigate services between health and social care systems (*n* = 22 reviews). This includes: • Addressing barriers to care for service users • Identifying appropriate services based on individual need • Facilitating communication between individuals and services • Signposting or connecting individuals with social care and community services	Patient navigator; Link worker; Care coordinator; Case manager; Health mediator; Intermediary; Social prescriber	(22, 23, 27, 29–31, 43–45, 47–50, 53–60, 62)
	Coordinating referrals, monitoring attendance, providing follow-up and reminding service users of upcoming activities (*n* = 11 reviews)	Patient navigator; Care coordinator; Case manager; Social prescriber	(22, 23, 29, 45, 47, 48, 54, 55, 57, 60, 62)
	Scheduling healthcare appointments with, or on behalf of, the servicer user (*n* = 9 reviews)	Patient navigator; Link worker; Social prescriber	(31, 45, 47, 48, 54, 56, 57, 60, 62)
Capacity building and supporting self-management (*n* = 20 reviews)	Education of the service user (*n* = 13 reviews). This includes: • Education of the health system • Education relevant to managing health conditions • Developing in self-management and health literacy	Patient navigator; Case manager; Care coordinator; Health mediator	(22, 23, 29, 45, 47, 48, 53–57, 59, 60)
	Providing motivational and emotional support, such as interpersonal support and coaching (*n* = 18 reviews)	Patient navigator; Link worker; Case manager; Care coordinator; Intermediary; Social prescriber	(22, 23, 29–31, 44, 45, 47–49, 53–58, 60, 62)
	Supporting goal setting processes (*n* = 11 reviews)	Patient navigator; Link worker; Intermediary	(23, 30, 31, 43–45, 47–49, 54, 57)
Personalised care and relationship building (*n* = 18 reviews)	Assessment of health and wellbeing needs (*n* = 13 reviews)	Patient navigator; Link worker; Care coordinator; Case manager; Health mediator; Intermediary; Social prescriber	(22, 23, 29–31, 47–50, 56, 58, 59, 62)
	Developing action plans, providing ongoing support and problem solving (*n* = 10 reviews)	Patient navigator; Care coordinator; Case manager; Link worker	(22, 23, 29, 44, 47, 48, 56–58, 60)
	Person-centred relational support (*n* = 13 reviews). This includes: • Advocating for service users with other care teams and services • Supporting service users who have their own action plans • Modelling health behaviours	Patient navigator; Link worker; Care coordinator; Case manager; Health mediator; Intermediary; Social prescriber	(22, 30, 47–51, 54, 56–59, 62)
Practical tasks (*n* = 17 reviews)	Accompanying service users to appointments or activities (*n* = 12 reviews)	Patient navigator; Link worker	(22, 23, 30, 31, 44, 45, 48, 50, 54–57)
	Delivering prescribed activities (*n* = 5 reviews)	Patient navigator; Link worker; Case manager	(27, 47, 48, 54, 55)
	Providing logistical support (*n* = 10 reviews). This includes: • Assisting with healthcare or social care related paperwork • Arranging transportation or childcare • Assisting with domestic tasks • Distributing exercise and food resources	Patient navigator; Link worker; Case manager; Social prescriber	(22, 30, 43, 47, 54–57, 60, 62)
	Acting as language interpreters (*n* = 3 reviews)	Patient navigator	(45, 47, 55)
Non-service user facing tasks (*n* = 15 reviews)	Identifying and developing connections with community and voluntary sector organisations (*n* = 10 reviews)	Patient navigator; Link worker; Care coordinator; Case manager; Health mediator; Social prescriber	(22, 23, 31, 43, 47, 55, 57–59, 62)
	Collaborating with health organisations and providers to improve coordination of care (*n* = 12 reviews)	Patient navigator; Link worker; Care coordinator; Case manager; Health mediator	(23, 31, 43, 45, 47, 48, 51, 53, 55, 57–59)
	Conducting outreach, through fundraising, community drop-in services, and advertising activities (*n* = 3 reviews)	Patient navigator; Link worker	(22, 31, 47)
	Managing and tracking client lists (*n* = 4 reviews)	Patient navigator; Link worker	(31, 48, 54, 55)
	Involvement in research or peer learning (*n* = 3 reviews). This includes: • Peer supervision and discussion groups • Providing education on relevant cultural practices to other health or social care providers	Patient navigator; Link worker; Case manager	(31, 47, 55)
Clinical tasks (*n* = 6 reviews)	Conducting assessments of health conditions, developing treatment plans, and involvement in clinical treatment delivery (*n* = 6 reviews)	Patient navigator; Care coordinator; Case manager	(23, 47, 52, 55, 58, 60)

Synthesis conducted using 23 included reviews that provided sufficient detail on tasks and responsibilities of care navigators. Reviews could report multiple task domains. Role category reported if at least one review of the role category described the task domain.

#### Tasks by role category

Tasks that were reported across all role categories in this umbrella review included helping service users navigate services between health and social care systems, assessment of health and wellbeing needs, and person-centred relational support. All other tasks varied among role categories (Table 2). A further comparison of tasks by role category was limited to 17 reviews that provided adequate detail and could be grouped by the two main role categories of *Link Workers* (*n* = 10 reviews) and *Patient Navigators* (*n* = 7 reviews). The frequencies in the data of other role categories (*Care Coordinator*, *Case Manager*, *Intermediary*, *Health Mediator*, and *Social Prescriber*) were too small for meaningful interpretation.

The core task shared by *Link Workers* and *Patient Navigators* was to assist service users to navigate services between health and social care systems. Both *Link Workers* and *Patient Navigators* were responsible for building the capacity of service users and enhancing self-management skills, typically through motivational and emotional support, and goal setting processes.

**Figure 4:**
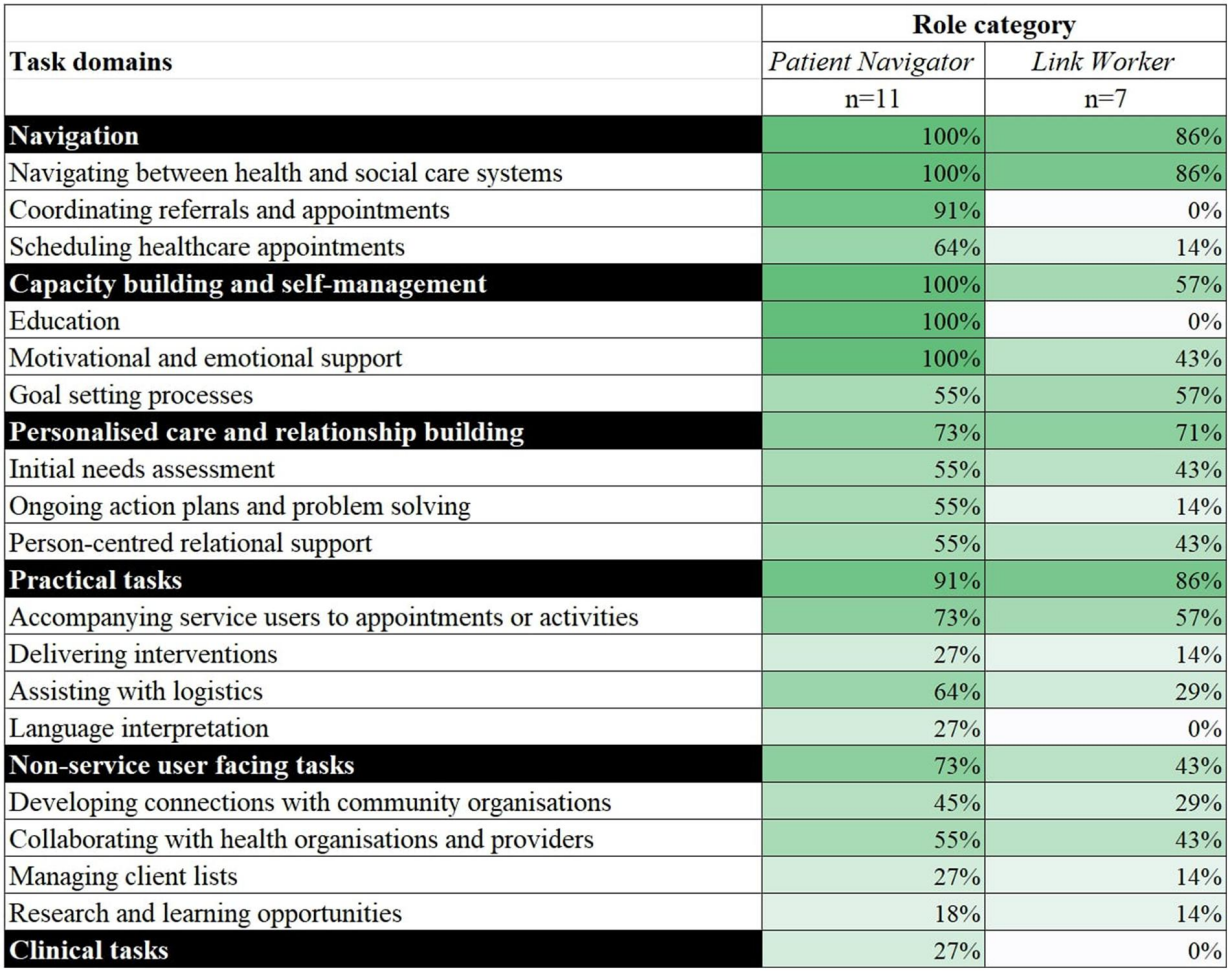
Differences in task domains between patient navigator and link worker categories. Role categories of *Care Coordinator*, *Case Manager*, *Intermediary*, *Health Mediator*, and *Social Prescriber* removed due to limited reporting of task domains (i.e., reported in ≤2 reviews). Percentage calculation: numerator is the number of reviews that discuss each task domain, denominator is number of reviews discussing each role that had sufficient data relevant to tasks and responsibilities. Colours assigned as a visual graded representation of percentages, from white (0%) to dark green (100%).

*Link Workers* and *Patient Navigators* shared all task domains, except for clinical tasks. Clinical tasks were only reported for *Patient Navigators* (*n* = 3) (23, 55, 60). Clinical tasks were not reported among *Link Workers,* and *Link Workers* did not provide education, coordination of referrals or appointments, nor did they act as interpreters, while *Patient Navigators* performed these tasks (Figure 4). Coordinating referrals and appointments was reported by 10 reviews focused on *Patient Navigators* (22, 23, 29, 45, 47, 48, 54, 55, 57, 60), and seven reviews on *Patient Navigators* described scheduling healthcare appointments on behalf of the service user (45, 47, 48, 54, 56, 57, 60), while only one review reported that *Link Workers* scheduled healthcare appointments (31). Three reviews (45, 47, 55) reported that *Patient Navigators* supported individuals with language interpretation, for example, in the context of coordinating appointments or assisting with paperwork to access health and social care (47). As depicted in Figure 4, *Patient Navigators* were more commonly involved in practical, specific tasks including clinical tasks needed by the client, whereas *Link Workers* mostly supported service navigation, linking and signposting services, and goal-setting.

## Discussion

Across 26 included reviews, 78 unique care navigator titles were identified, corresponding to seven role categories: *Patient Navigator*, *Link Worker*, *Care Coordinator*, *Case Manager*, *Social Prescriber*, *Intermediary*, and *Health Mediator*. Navigation programs were predominantly delivered in primary or community healthcare settings. Six broad task domains emerged from the analysis, with navigation, and capacity building and supporting self-management most frequently reported. *Patient Navigators* were more frequently reported to provide education to service users, undertake clinical tasks, and schedule healthcare appointments compared to *Link Workers.*

The large variability in the titles and role definitions utilised amongst the care navigation workforce introduces complexity into the way roles are understood by service users, practitioners and other professional staff, external stakeholders, and researchers. This creates difficulties in embedding these roles into healthcare and social care teams, including misunderstandings of role scope and potential task overlaps and duplication with existing roles. Health professionals who have a limited understanding of navigators' role scope, skills and capacity may refer patients or clients who are unlikely to benefit. For example, Brunton et al. reported that patients with complex needs were simply handed over to navigators even when there was uncertainty about the navigators' capacity and resources to manage complex patients (24). Complexity in role definition and highly variable training for the role may also limit the development of a unified professional identity for care navigators; emerging competency frameworks endeavour to bridge the gaps in programs and education pathways (10). To support navigator roles, learning communities of practice could be established to provide opportunities for professional development, sharing of professional knowledge and resources, while providing opportunities for peer support.

Although 78 unique titles were identified in this umbrella review, this is likely to be an underestimation of the true variety of role titles used, as a previous review that used a more limited scope (i.e., social prescribing only) identified just as many titles (21). This is likely due to the synthesis being limited to reviews, which may not capture all titles indicated in primary sources. In previous studies, attempts have been made to differentiate or consolidate navigation models and role titles (6, 47, 63, 64), particularly for roles of patient navigators, care coordinators, and case managers, but conclusions are often contradictory. The current umbrella review describes even greater complexity by presenting evidence that the titles of case manager, patient navigator and care coordinator may be used both interchangeably and as distinct roles. Moreover, despite available conventions in social prescribing schemes (65), variation in titles persists (30, 31). There is a paucity of evidence exploring how these titles are defined, decided upon, and understood by various stakeholders and most importantly by service users, however, a UK National Health Service (NHS) guideline asserts that these titles depend on local preferences of stakeholders developing and implementing navigator services (65).

The implementation of multiple models of navigation within different contexts, and the resulting complexity of titles, roles, and responsibilities, reflects the widespread recognition of integrated care and navigator roles as critical in addressing system fragmentation and increasing needs of service users (3, 9). Moving towards an international consensus on role nomenclature and core role functions and tasks across numerous navigation models of care would be helpful to ensure this workforce is appropriately understood by health and social care providers, service users, external stakeholders, and the individuals fulfilling the navigator role. Frameworks such as Lukersmith's case management taxonomy (66) could be leveraged to guide the development of a novel nomenclature and taxonomy to consolidate the myriad of roles titles, tasks and role scopes inconsistently described in the literature. The seven role categories and six task domains synthesised in this umbrella review provide a useful foundation for consolidating understanding on this emerging workforce. Based solely on the overlapping titles presented in Figure 3, *Care Coordinators, Case Managers, Health Mediators,* and *Patient Navigators* could potentially be conceptually combined into one group. However, tasks identified in this umbrella review differ between these four role categories, and a previous review has specifically delineated the difference between *Case Manager*s and *Patient Navigators* (47). Therefore, consolidating the roles that exist under these models of care would necessitate broader reconciliation of the theoretical underpinning of their implementation. Using the synthesis presented in our paper as a starting point, and leveraging existing frameworks, an international Delphi study could be undertaken to develop a consensus on the nomenclature and taxonomy for navigator roles that interface between health and social care systems.

A clearer pathway to standardisation exists for the role categories of *Link Worker* and *Social Prescriber*, as all reviews using these terms were focused on social prescribing (27, 30, 31, 42–44, 46, 50, 51, 62). “Link worker” is the generic title for navigators working under social prescribing schemes in the UK (65), and was more commonly used in included reviews. Using the “link worker” term to describe care navigators working in social prescribing schemes would enable consistency in future research and evidence synthesis. This is important for developing a deeper understanding of the workforce on a broad research or policy level, and is particularly important for developing professional identity and to demonstrate overall effectiveness of emerging roles. However, standardisation of the term “link worker” may remove the contextual nuance of existing role titles, which could be detrimental for implementing services on a local level. This is particularly relevant where programs have already been established, such as a program in the UK that implemented a “social prescribing coordinator” and the term is broadly known and accepted by local stakeholders (67). If this established program were to change the role title from “social prescribing coordinator” to “link worker” as suggested above, service users and collaborating health and social care professionals may experience greater confusion as an unintended consequence of standardisation. A middle ground for resolving this tension would be for existing role titles, or newly developed roles embedded in a certain context, to be clearly linked to a model of care (e.g., social prescribing) to ensure appropriate synthesis in research and evaluations, while maintaining contextual nuance.

The tasks and responsibilities of care navigators identified in this umbrella review align with previous syntheses and guidelines, which emphasise navigation or signposting to available services, self-management support, offering education, and engaging in relational support or advocacy for the service user (10, 47). The inclusion of a wide range of roles in this review allowed for a broader and more nuanced analysis of tasks and responsibilities across different roles rather than focusing on certain roles as reported in previous reviews (10, 30, 47, 55).

Key differences between *Patient Navigators* and *Link Workers* emerged on the task level. For example, within the navigation domain, *Link Workers* also rarely coordinated or scheduled care appointments for the service user, whereas this was often reported for *Patient Navigators*. This is unsurprising given that patient navigation was originally conceptualised to assist cancer patients in navigating through health services (4), with the initiation of referrals to services indicated as a core function (47). Link workers have been described in primary sources as providing coordination of referrals (68, 69); however, these referrals are generally to community-based activities rather than discrete appointments with health or social care practitioners, and may have been differently conceptualised and reported in the included reviews. Understanding the similarities and differences between the theory and objectives underpinning each model, and how these objectives are translated into responsibilities of navigators in practice, will assist in providing clarity as these models of care continue to evolve. In moving towards an international consensus on navigator roles and their boundaries, delineation of tasks and responsibilities should also allow for fluidity, given the often context-dependent nature of role implementation and a focus on individual needs.

In this umbrella review, paid navigators from all professional backgrounds were included, and around half of the included reviews focused on programs that were not specific to certain conditions (e.g., cancer). To bolster this unique and valued navigator workforce, there is a need to build a professional identity by developing and strengthening relevant navigator training, continuing education, peer support and communities of practice. For example, the UK National Academy for Social Prescribing provides structured training, resources and support for link workers while working with health and community organisations and leaders to embed link workers as an integral part of the health and social care system (70). Similar frameworks have also been established for case managers, which provide clear standards of practice for case managers (71), and highlight professional development as a core competency (72). A more general competency framework for care navigation has been developed in the UK which provides high-level guidelines for care navigators working across multiple models of care, including social prescribing and patient navigation (10). The development of this general care navigation framework acknowledges the overlapping functions of the navigation workforce, and aligns with other attempts in the UK to separate roles of care coordinator, social prescribing link worker, and health and wellbeing coach under the umbrella of personalised care services (8).

Providing structured training, standards of practice, and professional development for emerging roles may also support the effective embedding of navigator models in practice by further legitimising the navigator position and providing clarity of role purpose (24). The clarification of role boundaries and development of standards of practice may also enhance the safety of service users, such as through improving navigator competency in supporting individuals with complex needs and ensuring that service users understand who is delivering their care. The communication of role boundaries and purpose to other staff and stakeholders, such as referring providers or administrative staff, may also minimise inappropriate and potentially harmful use of navigation services, such as the referral of individuals with acute or emergency needs to social prescribing link workers (73).

On a research level, reporting worker titles and corresponding tasks and responsibilities consistently, would enable meaningful comparisons and interpretation of research and evaluation results across role categories, scopes of practice and models of care. Therefore, researchers and journal editors have a responsibility to encourage clear descriptions of role scopes, competencies and tasks, when publishing research related to the navigator workforce, to avoid confusion and to reduce the current complexity of reporting on this growing workforce.

### Strengths and limitations

This study adhered to PRISMA systematic review and JBI umbrella review reporting guidelines; the search strategy was also developed in consultation with two experienced clinical librarians (J Cullis, S Lewis). Reviews were included that focused on care navigator roles in any health setting, country, or population, which enabled a broad synthesis of titles and roles that exist across multiple contexts. However, only reviews written in English were included which may introduce language bias. Terminology used in the search strategy to identify navigator roles was also limited, and excluded certain terms such as community health worker that may have been relevant or implemented in low- or middle-income countries. However, given the sheer scope and variability in titles used within this research field, it was not feasible to screen all potential terms.

This umbrella review also only included reviews published within the last five years (2019–2024) to ensure that the most recent and relevant evidence was captured. This scope may have resulted in the exclusion of otherwise relevant reviews published prior to the search period [e.g., Carter et al. 2018 (28)] and may not capture the full scope of care navigator roles. A mitigating factor to this limitation is that included reviews often included primary sources that were published prior to 2019, meaning that the data extracted and synthesised from included reviews reflected navigator roles over a broad time period. Although no included reviews focused solely on care navigation delivered during the COVID-19 pandemic, some primary sources included details of adaptations and experiences of navigators in the pandemic (74–77). Future reviews could explore the impact of the COVID-19 pandemic on navigator roles and boundaries.

The synthesis in this umbrella review was also limited to findings reported in included reviews, as extracting from primary sources was beyond the scope of the study. Moreover, the role categories of *Care Coordinator, Case Manager, Intermediary, Health Mediator,* and *Social Prescriber* were each reported in ≤2 reviews, making it difficult to reliably determine whether a lack of data related to a task was due to the activity not being part of the role itself, or due to a lack of reporting at the review level. Consequently, these role categories were not included in detailed analysis that separated tasks by role category.

## Conclusion

The growing demand for care navigators to support individuals within health, social, and community care is reflected in the large variability of titles and role scopes identified in this umbrella review. However, this variability may create complexity and confusion for service users, health and social care providers, and researchers. Consolidating the understanding of these roles as implemented in various models of care, while clarifying expectations among service users and professional staff who interface with the navigator workforce would improve the embedding of these roles in health and social care systems and enhance effective service delivery. Developing professional identities and strategies to delineate role boundaries within health and social care services, alongside appropriate navigator training and workforce development, is needed to maximise the value that care navigators, coordinators, or link workers bring to our overstretched care systems.

## Data Availability

The original contributions presented in the study are included in the article/Supplementary Material, further inquiries can be directed to the corresponding author.

## Supplementary material

Supplementary File 1 Search strategy.

Supplementary Table 1 Quality appraisal using the JBI Checklist for Systematic Reviews.

Supplementary Table 2 Quality appraisal using the RAMESES Quality Standards.
