# Supplementary material for: Working between systems: an umbrella review of care navigator roles and responsibilities
Source: Front Health Serv. 2025 Oct 24;5:1632307. doi: 10.3389/frhs.2025.1632307 (PMC12592172; doi:10.3389/frhs.2025.1632307)
Supplement: Supplementary File 1 — Search strategy. [file Supplementaryfile1.docx]

**Supplementary Search Details**

***MEDLINE (Ovid)***

1 Patient Navigation/ 1122

2 health* navigat*.ab,kw,ti. 248

3 community navigat*.ab,kw,ti. 76

4 care navigat*.ab,kw,ti. 308

5 patient navigat*.ab,kw,ti. 1795

6 link work*.ab,kw,ti. 195

7 (social adj2 prescri*).ab,kw,ti. 581

8 care facilitat*.ab,kw,ti. 510

9 care coordinator*.ab,kw,ti. 1006

10 community-links practitioner*.ab,kw,ti. 4

11 health coach*.ab,kw,ti. 1506

12 health mediat*.ab,kw,ti. 322

13 clinical-community link*.ab,kw,ti. 25

14 or/1-13 6748

15 professional role/ or "scope of practice"/ 16673

16 exp education, professional/ 337235

17 exp Inservice Training/ 30096

18 exp Professional Competence/ 131946

19 Health Workforce/ 14705

20 (train* or educat* or accreditat* or experience* or qualif*).mp. 3107072

21 (scope or role* or competen* or task* or responsibili* or workload or position description or job description).mp. 4598775

22 ((support* or sustain*) adj2 (fund* or econom* or financ* or organis*)).mp. 26148

23 or/15-22 7127767

24 14 and 23 4244

25 limit 24 to (english language and yr="2019 -Current") 2374

26 limit 25 to "review articles" 226

27 review*.ab,ti. 2920483

28 25 and 27 440

29 26 or 28 491

***Embase***

1 health* navigat*.ab,kw,ti. 384

2 community navigat*.ab,kw,ti. 116

3 care navigat*.ab,kw,ti. 512

4 patient navigat*.ab,kw,ti. 3205

5 link work*.ab,kw,ti. 243

6 (social adj2 prescri*).ab,kw,ti. 735

7 care facilitat*.ab,kw,ti. 720

8 care coordinator*.ab,kw,ti. 1776

9 community-links practitioner*.ab,kw,ti. 4

10 health coach*.ab,kw,ti. 2197

11 health mediat*.ab,kw,ti. 351

12 clinical-community link*.ab,kw,ti. 22

13 or/1-12 9918

14 professional role/ or "scope of practice"/ 40613

15 exp education, professional/ 11951

16 exp Inservice Training/ 16155

17 exp Professional Competence/ 34847

18 Health Workforce/ 4692

19 (train* or educat* or accreditat* or experience* or qualif*).mp. 4290263

20 (scope or role* or competen* or task* or responsibili* or workload or position description or job description).mp. 5647496

21 ((support* or sustain*) adj2 (fund* or econom* or financ* or organis*)).mp. 32628

22 or/14-21 9182317

23 13 and 22 6506

24 limit 23 to (english language and yr="2019 -Current") 3384

25 limit 24 to (conference abstract or conference paper) 1345

26 24 not 25 2039

27 review*.ab,ti. 3724585

28 26 and 27 381

29 limit 28 to "review" 161

30 28 or 29 381

31 26 not 30 1658

***PsycINFO***

1 health* navigat*.mp. 117

2 community navigat*.mp. 45

3 care navigat*.mp. 96

4 patient navigat*.mp. 546

5 link work*.mp. 112

6 (social adj2 prescri*).mp. 337

7 care facilitat*.mp. 165

8 care coordinator.mp. 176

9 community-links practitioner.mp. 1

10 health coach*.mp. 430

11 health mediat*.mp. 216

12 clinical-community link*.mp. 10

13 or/1-12 2179

14 (train* or educat* or accreditat* or experience* or qualif*).mp. 1719632

15 (scope or role* or competen* or task* or responsibili* or workload or position description or job description).mp. 1389484

16 ((support* or sustain*) adj2 (fund* or econom* or financ* or organis*)).mp. 9063

17 exp professional certification/ or exp professional competence/ or exp professional development/ 76138

18 exp inservice training/ 3461

19 exp professional role/ 4225

20 or/14-19 2659116

21 13 and 20 1444

22 limit 21 to (english language and yr="2019 -Current") 545

23 "review".ti,ab,hw,id. 448849

24 22 and 23 72

***CINAHL***

| S1 | (MH “Patient Navigation”) |
| --- | --- |
| S2 | AB "health* navigat*” |
| S3 | AB "community navigat*” |
| S4 | AB "care navigat*” |
| S5 | AB "patient navigat*” |
| S6 | AB "link work*” |
| S7 | AB social N2 prescri* |
| S8 | AB "care facilitat*” |
| S9 | AB "care coordinator*” |
| S10 | AB "community-links practitioner*” |
| S11 | AB "health coach*” |
| S12 | AB "health mediat*” |
| S13 | AB "clinical-community link*” |
| S14 | S1 OR S2 OR S3 OR S4 OR S5 OR S6 OR S7 OR S8 OR S9 OR S10 OR S11 OR S12 OR S13 |
| S15 | (MH "Professional Role+") |
| S16 | (MH "Scope of Practice+") |
| S17 | (MH "Professional Competence+") |
| S18 | (MH "Staff Development+") |
| S19 | ""inservice training"" |
| S20 | (MH "Health Personnel+") |
| S21 | train OR educat* OR accreditat* OR experienc* OR qualif* |
| S22 | "scope OR role* OR competen* OR task* OR responsibili* OR workload OR "position description" OR "job description"" OR (MH "Work Engagement+") OR (MH "Workload") OR (MH "Job Description") OR (MH "Job Experience") OR (MH "Job Characteristics") OR (MH "Mentorship") OR (MH "Cross Training") |
| S23 | “"(support* OR sustain*) N2 (fund* OR econom* OR financ* OR organis*)"” |
| S24 | S15 OR S16 OR S17 OR S18 OR S19 OR S20 OR S21 OR S22 OR S23 |
| S25 | S14 AND S24 |
| S26 | S14 AND S24  Limiters - Publication Date: 20190101-20241231 Expanders - Apply equivalent subjects Narrow by Language: - english Search modes - Boolean/Phrase |
| S27 | S14 AND S24  Limiters - Publication Date: 20190101-20241231; Publication Type: Review Expanders - Apply equivalent subjects Search modes - Boolean/Phrase |
| S28 | S14 AND S24  Limiters - Publication Date: 20190101-20241231; Publication Type: Systematic Review Expanders - Apply equivalent subjects Search modes - Boolean/Phrase |
| S29 | AB review* |
| S30 | S26 AND S29 |
| S31 | S27 OR S28 OR S30 |
| S32 | S26 NOT S31 |

***Scopus***

( TITLE-ABS-KEY ( "health* navigat*" ) OR TITLE-ABS-KEY ( "community navigat*" ) OR TITLE-ABS-KEY ( "care navigat*" ) OR TITLE-ABS-KEY ( "patient navigat* " ) OR TITLE-ABS-KEY ( "link work*" ) OR TITLE-ABS-KEY ( "social W/2 prescrib*" ) OR TITLE-ABS-KEY ( "care facilitat*" ) OR TITLE-ABS-KEY ( "care coordinator*" ) OR TITLE-ABS-KEY ( "community-links practitioner*" ) OR TITLE-ABS-KEY ( "health coach*" ) OR TITLE-ABS-KEY ( "health mediat*" ) OR TITLE-ABS-KEY ( "clinical-community link*" ) ) AND ( TITLE-ABS-KEY ( train* ) OR TITLE-ABS-KEY ( educat* ) OR TITLE-ABS-KEY ( accreditat* ) OR TITLE-ABS-KEY ( experienc* ) OR TITLE-ABS-KEY ( qualif* ) OR TITLE-ABS-KEY ( role* ) OR TITLE-ABS-KEY ( scope* ) OR TITLE-ABS-KEY ( competen* ) OR TITLE-ABS-KEY ( task* ) OR TITLE-ABS-KEY ( responsibili* ) OR TITLE-ABS-KEY ( workload ) OR TITLE-ABS-KEY ( "position description" ) OR TITLE-ABS-KEY ( "job description" ) OR TITLE-ABS-KEY ( ( support* OR sustain* ) W/2 ( fund* OR econom* OR financ* OR organis* ) ) ) AND PUBYEAR > 2018 AND PUBYEAR < 2026 AND ( TITLE-ABS-KEY ( review* ) ) AND ( LIMIT-TO ( LANGUAGE , "English" )
