# Supplementary material for: Working between systems: an umbrella review of care navigator roles and responsibilities
Source: Front Health Serv. 2025 Oct 24;5:1632307. doi: 10.3389/frhs.2025.1632307 (PMC12592172; doi:10.3389/frhs.2025.1632307)
Supplement: Supplementary Table 1 — Quality appraisal using the JBI Checklist for Systematic Reviews. [file Table1.docx]

**Supplementary Table 1. Quality appraisal of included reviews using the JBI Checklist for Systematic Reviews**

|  | **1. Is the review question clearly and explicitly stated?** | **2. Were the inclusion criteria appropriate for the review question?** | **3. Was the search strategy appropriate?** | **4. Were the sources and resources used to search for studies adequate?** | **5. Were the criteria for appraising studies appropriate?** | **6. Was critical appraisal conducted by two or more reviewers independently?** | **7. Were there methods to minimize errors in data extraction?** | **8. Were the methods used to combine studies appropriate?** | **9. Was the likelihood of publication bias assessed?** | **10. Were recommendations for policy and/or practice supported by the reported data?** | **11. Were the specific directives for new research appropriate?** |
| --- | --- | --- | --- | --- | --- | --- | --- | --- | --- | --- | --- |
| **Choi (2021)** | Yes | Yes | Yes | Yes | NA | NA | Yes | Yes | NA | Yes | Yes |
| **Desveaux (2019)** | Yes | Yes | Yes | Yes | Yes | Unclear | Yes | Yes | NA | Yes | Yes |
| **Doucet (2022)** | No | Yes | Yes | Yes | NA | NA | Yes | Yes | No | Yes | NA |
| **Ebrahimoghli (2023)** | Yes | Yes | Yes | Unclear | Yes | Yes | Yes | Yes | No | Yes | NA |
| **Harris (2023)** | Yes | Yes | Yes | Yes | Yes | Yes | Unclear | Unclear | NA | Yes | Yes |
| **Kelly (2019)** | Yes | Yes | Yes | Yes | NA | NA | No | Unclear | No | Yes | Yes |
| **Koenig (2021)** | Yes | Yes | Yes | Yes | NA | NA | Yes | Yes | NA | Yes | Yes |
| **Kokorelias (2024)** | No | Yes | Yes | Yes | NA | NA | Yes | Yes | No | Yes | Yes |
| **Linceviciute (2023)** | Yes | Yes | Yes | Unclear | Yes | Yes | Yes | Yes | No | Yes | NA |
| **Mullen (2023)** | No | Unclear | Yes | Yes | NA | NA | Yes | Yes | No | Yes | NA |
| **O’Grady (2024)** | Yes | Yes | Yes | Yes | NA | NA | Yes | Yes | NA | Yes | Yes |
| **Osterholm (2023)** | Yes | Unclear | Yes | Yes | Yes | Yes | Yes | Yes | NA | Unclear | Unclear |
| **Rankin (2022)** | Yes | Yes | Yes | Yes | NA | NA | No | Yes | NA | Yes | Yes |
| **Rapo (2023)** | Yes | Yes | No | Yes | Yes | Yes | No | Yes | No | Yes | Yes |
| **Richard (2022)** | Yes | Yes | Yes | Yes | NA | NA | No | Yes | NA | Yes | Yes |
| **Roland (2022)** | Yes | Yes | Yes | Yes | Yes | Yes | Yes | Yes | NA | Yes | Yes |
| **Roland (2020)** | Yes | Yes | Yes | Yes | Yes | Yes | Yes | Yes | NA | Yes | Yes |
| **Rothe (2022)** | Yes | Yes | Yes | Yes | No | No | No | Unclear | No | Yes | Unclear |
| **Sandhu (2022)** | Yes | Yes | Yes | Yes | NA | NA | Yes | Yes | NA | Yes | Yes |
| **Shockney (2021)** | Yes | Yes | Yes | Yes | No | No | No | No | NA | Yes | Yes |
| **Stretton (2022)** | Yes | Yes | Yes | Yes | NA | NA | No | Yes | NA | Yes | Yes |
| **Surugiu (2023)** | No | Yes | Unclear | Yes | NA | NA | Unclear | Unclear | NA | Yes | Yes |
| **Yadav (2024)** | Yes | Yes | Yes | Yes | NA | NA | Yes | Yes | NA | Yes | Yes |
| **Zhang (2021)** | Yes | Yes | Yes | Yes | Yes | No | Yes | Yes | No | Yes | Yes |

NA=Not applicable
