# Supplementary material for: Working between systems: an umbrella review of care navigator roles and responsibilities
Source: Front Health Serv. 2025 Oct 24;5:1632307. doi: 10.3389/frhs.2025.1632307 (PMC12592172; doi:10.3389/frhs.2025.1632307)
Supplement: Supplementary Table 2 — Quality appraisal using the RAMESES Quality Standards. [file Table2.docx]

**Supplementary Table 2. Quality appraisal of included reviews using the RAMESES Quality Standards for Realist Syntheses and Meta-Narrative Reviews**

|  | **The research topic is appropriate for a realist approach** | **The research question is constructed in such a way as to be suitable for realist synthesis** | **The review demonstrates understanding and application of realist philosophy and realist logic which underpins a realist analysis** | **The review question is sufficiently and appropriately focussed** | **An initial realist programme theory is identified and developed** | **The search process is such that it would identify data to enable the review team to develop, refine and test programme theory or theories** | **The selection and appraisal process ensures that sources relevant to the review containing material of sufficient rigour to be included are identified.** | **The data extraction process captures the necessary data to enable a realist review** | **The realist synthesis is reported using the items listed in the RAMESES Reporting standard for realist syntheses** |
| --- | --- | --- | --- | --- | --- | --- | --- | --- | --- |
| **Calderon-Larranaga (2021)** | Good | Adequate | Good | Excellent | Excellent | Good | Adequate | Adequate | Excellent |
| **Tierney (2020)** | Excellent | Adequate | Good | Excellent | Excellent | Good | Good | Good | Excellent |

Note. Response options are: Inadequate, Adequate, Good, Excellent.
